# Efficacy and safety of pharmacotherapy for cancer cachexia: A systematic review and network meta‐analysis

**DOI:** 10.1002/cam4.70166

**Published:** 2024-09-03

**Authors:** Hao Chen, Masashi Ishihara, Hiroki Kazahari, Ryusuke Ochiai, Shigeru Tanzawa, Takeshi Honda, Yasuko Ichikawa, Nobuyuki Horita, Hisashi Nagai, Kiyotaka Watanabe, Nobuhiko Seki

**Affiliations:** ^1^ Department of Oncology Teikyo University School of Medicine Tokyo Japan; ^2^ Department of Pulmonology Yokohama City University Hospital Yokohama Japan; ^3^ Department of Chemotherapy Yokohama City University Hospital Yokohama Japan; ^4^ Graduate School of Human and Environmental Studies Tokai University Tokyo Japan

**Keywords:** anamorelin, body weight, cancer cachexia, network meta‐analysis, olanzapine

## Abstract

**Background:**

Cancer cachexia affects more than half of all cancer patients, reducing survival rates. Evidence‐based approaches are urgently needed to optimize treatment.

**Methods:**

A systematic review and network meta‐analysis were conducted to assess the effectiveness and safety of different pharmacotherapies for cancer cachexia. Three databases (PubMed, Cochrane Library, and Web of Science) were searched for the period from January 1, 2000, to March 20, 2024. The netmeta package in R software was used to calculate the pooled effect, employing a random effects model.

**Results:**

Seven placebo‐controlled randomized trials involving 1421 patients were analyzed. Pairwise analysis showed that body weight increases were 4.6 kg (95% confidence interval [CI] 0.83–8.37 kg) for olanzapine, 3.82 kg (95% CI 0.73–6.91 kg) for espindolol (20 mg), 2.36 kg (95% CI 1.84–2.89 kg) for anamorelin (100 mg), and 1.31 kg (95% CI 0.42–2.19 kg) for anamorelin (50 mg). In terms of safety profiles, olanzapine demonstrated the lowest odds ratio when compared to placebo, at 0.26 (95% CI 0.07–0.94), followed by anamorelin (50 mg) at 0.86 (95% CI 0.30–2.48), and anamorelin (100 mg) at 0.89 (95% CI 0.42–1.88). However, network meta‐analysis could not confirm the superiority of olanzapine over anamorelin in terms of efficacy and safety.

**Conclusion:**

Both olanzapine and anamorelin are useful in improving body weight in patients with cancer cachexia. Personalization may be helpful for different patients.

## 1 INTRODUCTION

Cancer cachexia is a multifactorial syndrome characterized by ongoing loss of skeletal muscle mass, with or without loss of fat mass, that cannot be fully reversed by conventional nutritional support and leads to progressive functional impairment.^1^ Half of all cancer patients experience cachexia. While the prevalence varies across cancer types, cachexia is most commonly associated with advanced disease stages, with the prevalence exceeding 80% in the last weeks of life.^2^ The condition not only significantly diminishes quality of life (QOL), but also is associated with poor treatment outcomes and reduced survival rates.^3^ Despite the severity of this condition, cancer cachexia remains underdiagnosed and undertreated, partly due to a lack of consensus on the definition and the complex pathophysiology involving inflammatory and metabolic imbalances triggered by tumor factors and host responses.^1^, ^4^

Over the years, the pharmacotherapy of cancer cachexia has evolved, moving from solely palliative care approaches to more targeted therapies aimed at the underlying mechanisms.^5^ Pharmacological interventions have expanded from nutritional supplements and appetite stimulants to include agents that modulate inflammation, metabolism, and muscle protein degradation.^6^ Among these are progesterone analogs, corticosteroids and, more recently, agents targeting specific cytokines and metabolic pathways implicated in cachexia. Despite such advances, the effectiveness of these treatments varies widely among patients, and evidence‐based guidelines to optimize therapeutic strategies remain urgently needed.^7^

In the absence of a definitive pharmacological solution for cancer cachexia, agents like anamorelin and olanzapine have emerged as significant.^8^, ^9^ Anamorelin is a ghrelin receptor agonist that effectively stimulates appetite and increases lean body mass, particularly in patients with non‐small cell lung cancer, by mimicking the hunger hormone ghrelin. Ghrelin analogs worked through an increase in growth hormone. Growth hormone may increase muscle mass without improving function.^10^, ^11^, ^12^ Olanzapine was initially used in psychiatry, but has shown promise in oncology for its appetite‐boosting and weight maintenance effects through neurotransmitter modulation.^13^ Additional treatments include megestrol acetate for appetite enhancement, corticosteroids for symptom relief, and fish oil supplements for muscle mass support and anti‐inflammatory effects, collectively contributing to the evolution of the pharmacotherapeutic landscape aimed at improving QOL in cachexia patients.^14^, ^15^

Despite numerous randomized controlled trials (RCTs) investigating various treatments for cancer cachexia, significant uncertainty remains regarding the most effective therapy. This lack of clarity can be attributed to heterogeneity in study populations, interventions, and outcomes assessed, making definitive conclusions difficult to draw from individual studies. Furthermore, direct comparisons between many of these treatments are scarce, leaving clinicians with limited guidance on choosing the optimal treatment strategy for patients. This study aimed to address this gap through a network meta‐analysis, allowing for the indirect comparison of multiple interventions across studies. This analysis seeks to identify the most effective treatments for cancer cachexia by synthesizing existing evidence as a path to informing clinical practice and guiding future research directions.

## 2 METHODS

The protocol was structured according to the Preferred Reporting Items for Systematic Reviews and Meta‐Analyses (PRISMA) statement.^16^ This study was registered on University Hospital Medical Information Network (ID: UMIN000053858).^17^ The need to obtain institutional review board approval was waived because of the systematic review nature of the research. H.C. and M.I. independently performed the search process and data extraction steps, and subsequently developed a consensus position.

### 2.1 Data search

Three major databases (PubMed, Cochrane Library, and Web of Science) were searched from January 1, 2000 to March 20, 2024 to identify candidate articles using terms referring to the strategies with patients (“cancer cachexia” or “cancer‐related anorexia”), intervention (“treatment” or “pharmacotherapy”), and control (“placebo” or “randomized trial”). Three review authors, H.C., M.I., and N.H., searched for additional eligible articles by checking the reference lists of included studies.

### 2.2 Inclusion and exclusion criteria

The search aimed to identify randomized clinical trials comparing a pharmacotherapy with placebo. Inclusion criteria were: (1) patients diagnosed with cancer cachexia; (2) outcomes identified after more than 1 month; and (3) randomized trials involving patients ≥16 years old. Exclusion criteria encompassed: (1) studies with supplementary additions; (2) samples from single‐arm studies with fewer than 10 cases; or (3) Phase I or Phase II studies. Both short articles and conference abstracts were considered.

### 2.3 Outcomes

Improved lean body mass was intended as the primary outcome, but that information was not available in all included studies. As a result, gain of body weight (BW) was selected as the primary outcome, and the odds ratio (OR) of adverse events. of Grade ≥3 was considered as the secondary outcome, according to the Common Terminology Criteria for Adverse Events by the National Cancer Institute.^18^

### 2.4 Statistics

Mean difference and standard deviation after each treatment arm were used to carry out a meta‐analysis to compare the efficacy of pharmacotherapy. ORs for adverse events of Grade ≥3 in each arm were calculated to allow comparison of the safety of pharmacotherapies. A least‐squares approach, random model network meta‐analysis, and pairwise meta‐analysis were performed using the netmeta command in the netmeta package, within R version 4.3.2 software (Gun project, Vienna).^19^ The *I* ^2^ statistic for heterogeneity was interpreted as follows: 0%, no heterogeneity; 0%–30%, weak heterogeneity; 30%–50%, moderate heterogeneity; 50%–75%, substantial heterogeneity; and 75%–100%, considerable heterogeneity. The Cochrane risk‐of‐bias tool for randomized trials was applied to evaluate the quality of randomized trial studies.^20^

## 3 RESULTS

### 3.1 Study search

A total of 1980 studies were collected after the search strategy was implemented across the three main databases (Figure S1). After removing duplicates and conducting a first‐round screening by title and abstract, 35 studies remained for full article screening. Ultimately, seven studies encompassing 1421 patients were included in the final network meta‐analysis. Characteristics of the included studies are shown in Table 1.^9^, ^21^, ^22^, ^23^, ^24^, ^25^, ^26^ Two studies identified the efficacy of anamorelin in non‐small cell lung cancer (NSCLC), while the remaining five studies evaluated the efficacies of enobosarm, mirtazapine, pentoxifylline, olanzapine, and espindolol, each in multiple cancer types. A BW decrease exceeding 5% is the most frequently used criterion for cancer cachexia, and changes in BW or lean body mass (LBM) were commonly set as primary outcomes.

**TABLE 1:** Characteristics of studies included in the network meta‐analysis.

Study	Country	Cases	Cancers	BW criteria	Primary outcome	Treatment	Duration
Currow et al.^21^	International	703	NSCLC	>5% BW loss or BMI <20 kg/m^2^	BW	Anamorelin	24 weeks
Dobs et al.^22^	International	159	Not specified	BMI <35 kg/m^2^ and >2% WB loss	LBM	Enobosarm	113 days
Hunter et al.^23^	Egypt	100	Not specified	>5% BW loss or BMI <20 kg/m^2^ and >2% BW loss	Appetite	Mirtazapine	8 weeks
Mehrzad et al.^24^	Iran	70	Not specified	>5% BW loss	BW, etc.	Pentoxifylline	2 months
Sandhya et al.^9^	India	124	Not specified	BW not specified	BW	Olanzapine	14 weeks
Coats et al.^25^	International	87	NSCLC or CRC	>5% BW loss, or BMI <20 kg/m^2^, or ongoing BW loss	BW	Espindolol	16 weeks
Takayama et al.^26^	Japan	178	NSCLC	>5% BW loss	LBM	Anamorelin	16 weeks

Abbreviations: BMI, body mass index; BW, body weight; CRC, colorectal cancer; LBM, lean body mass; NSCLC, non‐small cell lung cancer.

### 3.2 Efficiency of pharmacotherapy for cancer cachexia

**FIGURE 1:**
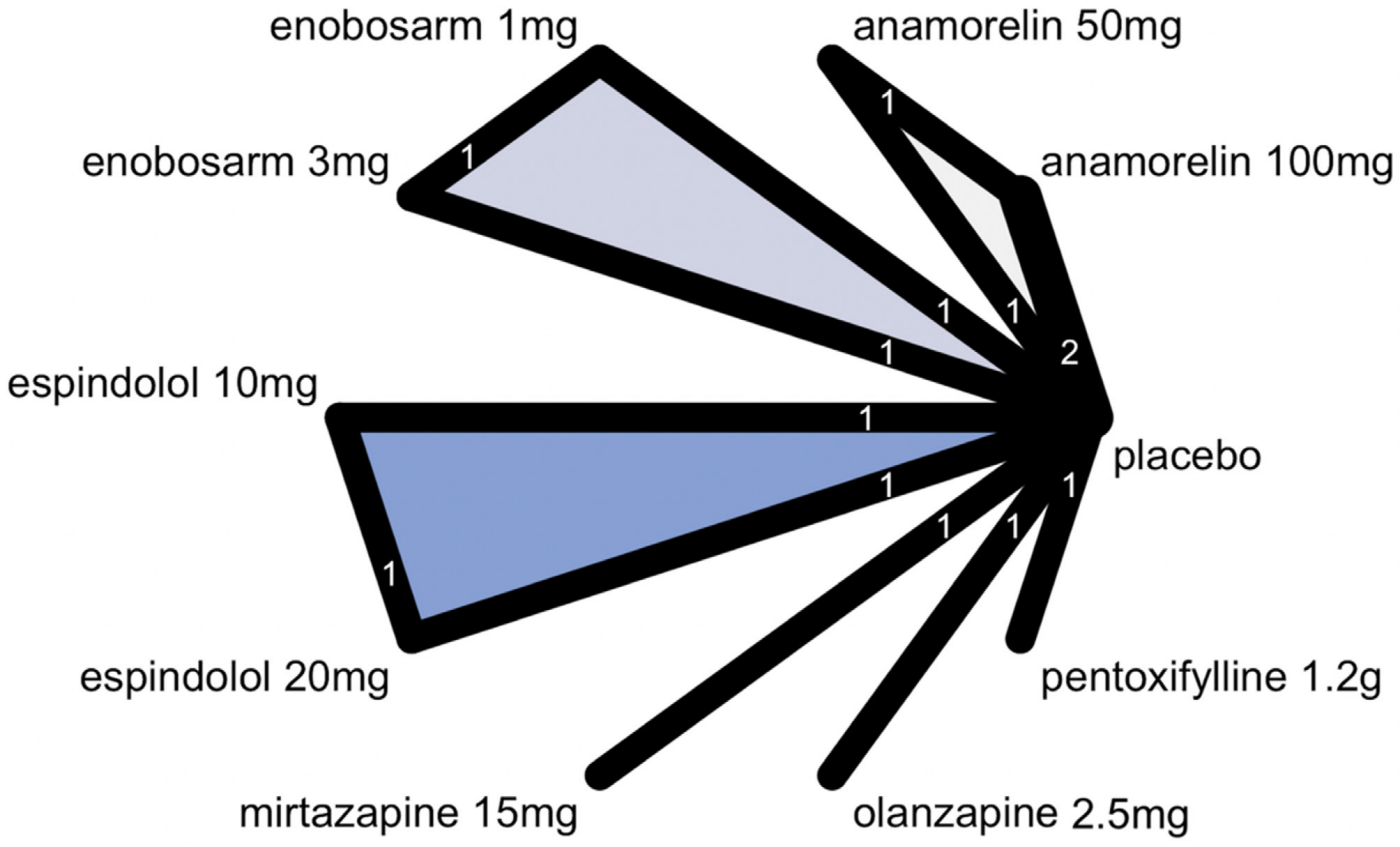
Network graph of studies included in the network meta‐analysis.

**FIGURE 2:**
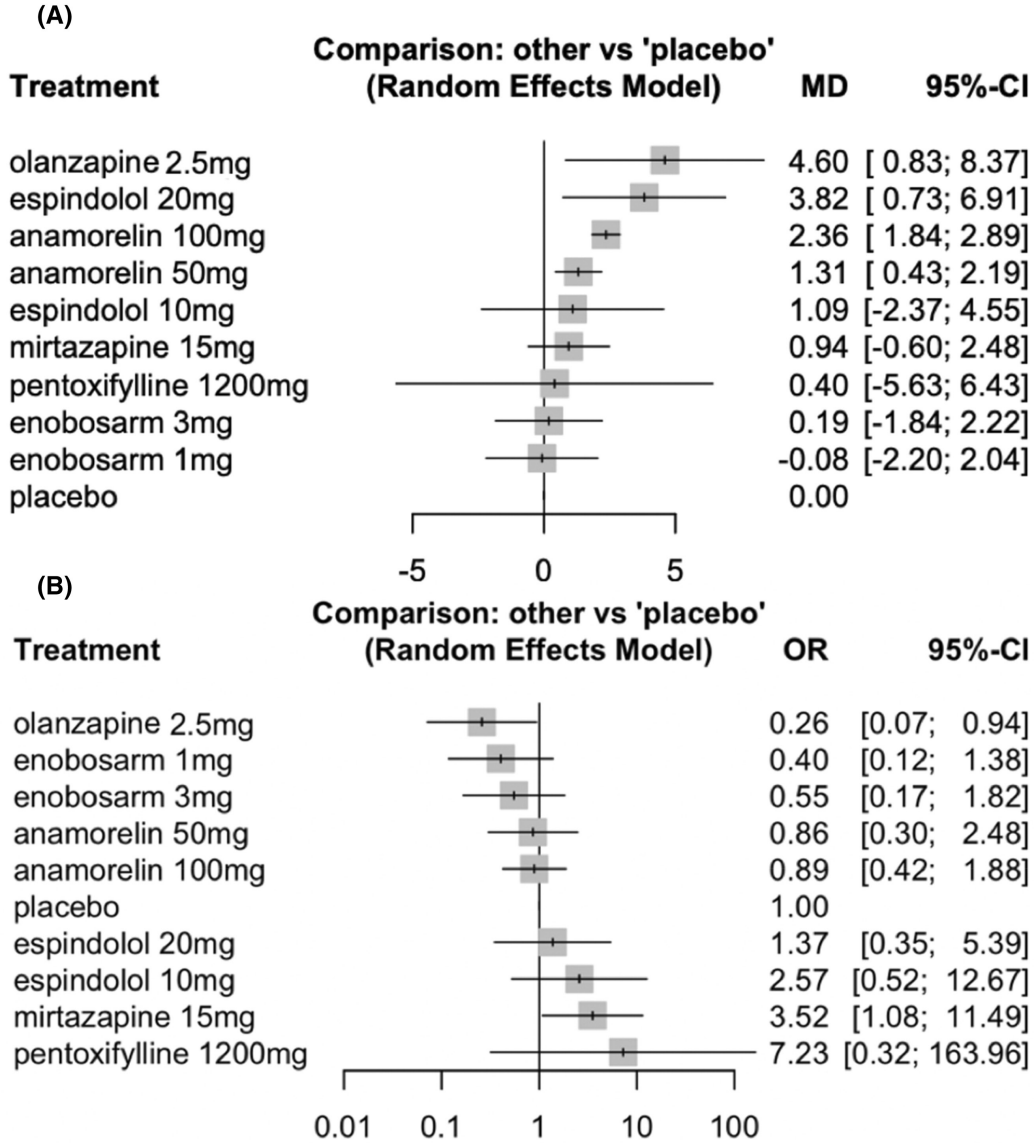
Effect of different treatments of weight gain and adverse events compared with placebo. (A) Different pharmacotherapies for improving body weight and (B) The safety of pharmacotherapy in relation to Grade 3 or higher adverse events. MD, mean difference; CI, confidence interval; OR, odds ratio.

The network graph of studies included in the network meta‐analysis is depicted in Figure 1, indicating the absence of direct comparisons between different medications. Anamorelin, enobosarm, and espindolol were evaluated for the efficacy of low and high doses. Results from pairwise meta‐analysis using a random effects model demonstrated the efficacy of different pharmacotherapies for improving BW (Figure 2). Four medications were identified as effective in improving BW: olanzapine, with a BW increase of 4.6 kg (95% confidence interval [CI] 0.83–8.37); espindolol (20 mg), with an increase of 3.82 kg (95% CI 0.73–6.91); anamorelin (100 mg), with an increase of 2.36 kg (95% CI 1.84–2.89); and anamorelin (50 mg), with an increase in 1.31 kg (95% CI 0.42–2.19).

**FIGURE 3:**
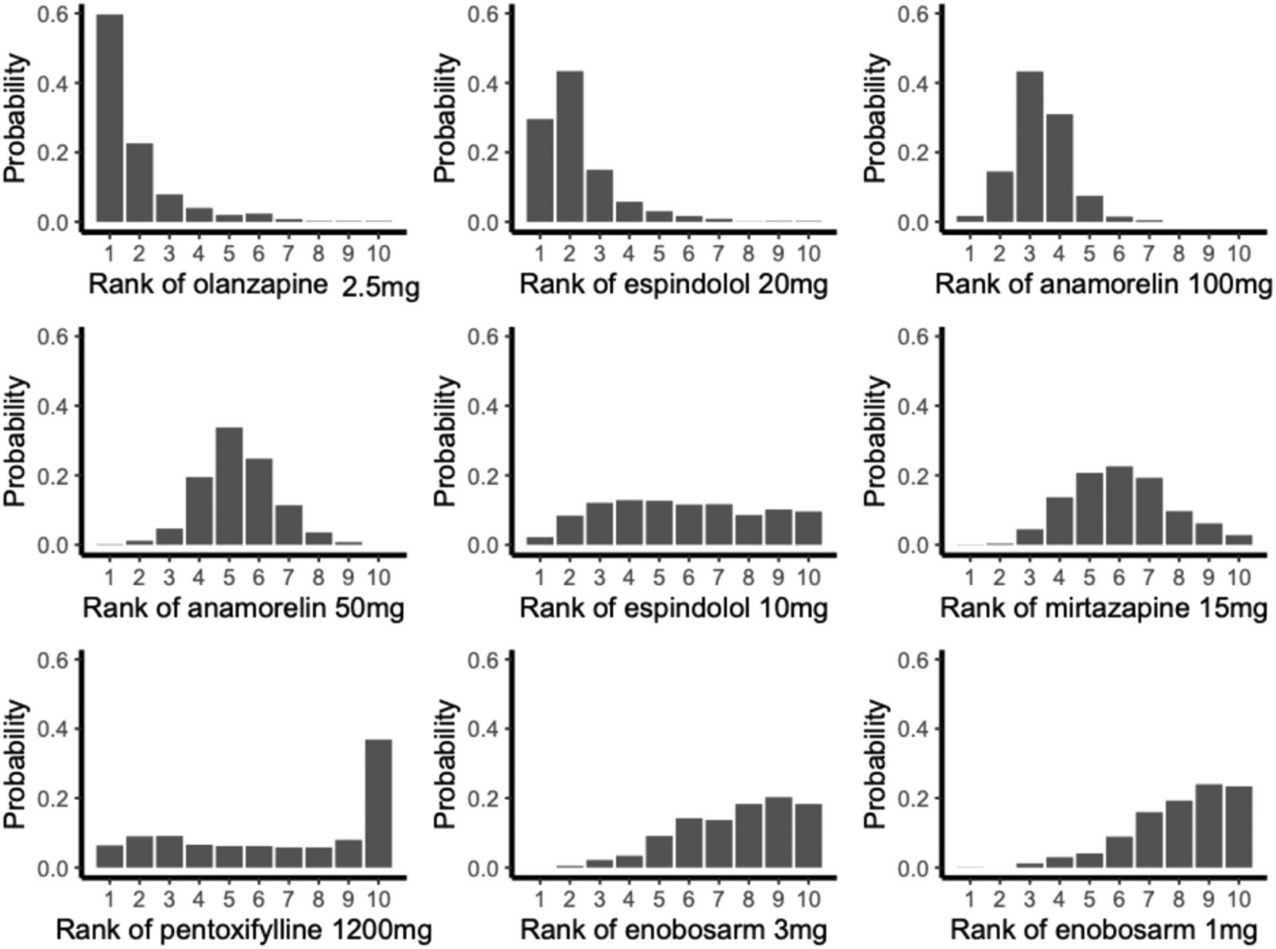
Rank of possibility of each treatment based on 1000 simulations.

The ranking of different treatments, based on 1000 simulations, is presented in Figure 3. The sequence of efficacy in decreasing order of magnitude was as follows: olanzapine (2.5 mg), espindolol (20 mg), anamorelin (100 mg), anamorelin (50 mg), espindolol (10 mg), mirtazapine (15 mg), pentoxifylline (1200 mg), enobosarm (3 mg), and enobosarm (1 mg). Outcomes from pairwise and network meta‐analyses are detailed in Table 2. The efficacy of olanzapine resulted in a BW increase in 0.78 kg (95% CI −4.09 to 5.65) compared with espindolol (20 mg), 2.24 kg (95% CI −1.57 to 6.04) compared with anamorelin (100 mg), and 3.29 kg (95% CI −0.58 to 7.16) compared with anamorelin (50 mg).

**TABLE 2:** League table of pairwise and network meta‐analyses by random model.

Olanz									4.6 (0.83, 8.37)
0.78 (−4.09, 5.65)	Espin_h			2.73 (0.25, 5.21)					3.82 (0.73, 6.91)
2.24 (−1.57, 6.04)	1.46 (−1.68, 4.59)	Anamo_h	1.30 (0.27, 2.33)						2.36 (1.84, 2.89)
3.29 (−0.58, 7.16)	2.51 (−0.71, 5.72)	1.05 (0.15, 1.95)	Anamo_l						1.5 (0.55, 2.45)
3.51 (−1.61, 8.63)	2.73 (0.25, 5.21)	1.27 (−2.23, 4.77)	0.22 (−3.35, 3.79)	Espin_l					1.09 (−2.37, 4.55)
3.66 (−0.41, 7.73)	2.88 (−0.57, 6.33)	1.42 (−0.2, 3.05)	0.37 (−1.4, 2.15)	0.15 (−3.64, 3.94)	Mirta				0.94 (−0.6, 2.48)
4.2 (−2.91, 11.3)	3.42 (−3.35, 10.2)	1.96 (−4.09, 8.02)	0.91 (−5.18, 7.01)	0.69 (−6.26, 7.64)	0.54 (−5.68, 6.76)	Pento			0.4 (−5.63, 6.43)
4.41 (0.13, 8.69)	3.63 (−0.07, 7.33)	2.17 (0.08, 4.27)	1.12 (−1.09, 3.33)	0.9 (−3.11, 4.91)	0.75 (−1.8, 3.3)	0.21 (−6.15, 6.57)	Enobo_h	0.27 (−1.83, 2.4)	0.19 (−1.84, 2.22)
4.68 (0.36, 9)	3.9 (0.15, 7.65)	2.44 (0.26, 4.63)	1.39 (−0.9, 3.69)	1.17 (−2.89, 5.23)	1.02 (−1.6, 3.64)	0.48 (−5.91, 6.87)	0.27 (−1.83, 2.37)	Enobo_l	−0.08 (−2.2, 2.04)
4.6 (0.83, 8.37)	3.82 (0.73, 6.91)	2.36 (1.84, 2.89)	1.31 (0.43, 2.19)	1.09 (−2.37, 4.55)	0.94 (−0.6, 2.48)	0.4 (−5.63, 6.43)	0.19 (−1.84, 2.22)	−0.08 (−2.2, 2.04)	Place

*Note*: Olan, olanzapine 2.5 mg; Espin_h, espindolol 20 mg; Anamo_h, anamorelin 100 mg; Anamo_l, anamorelin 50 mg; Espin_l, espindolol 10 mg; Mirta, mirtazapine; Pento, pentoxifylline 1200 mg; Enobo_h, enobosarm 3 mg; Enobo_l, enobosarm 1 mg; Place, placebo.

### 3.3 Safety of pharmacotherapy in cancer cachexia

The safety of pharmacotherapy for Grade ≥3 adverse events was assessed through pairwise comparisons (Figure 2B). Olanzapine demonstrated the lowest OR, at 0.26 (95% CI 0.07–0.94) when compared to placebo, followed by enobosarm 1 mg with an OR of 0.40 (95% CI 0.12–1.38), enobosarm 3 mg with an OR of 0.55 (95% CI 0.17–1.82), anamorelin (50 mg) with an OR of 0.86 (95% CI 0.30–2.48), and anamorelin (100 mg) with an OR of 0.89 (95% CI 0.42–1.88). Safety rankings for the different treatments, based on 1000 simulations, are presented in Figure S3, and aligned with the results of pairwise analysis.

Outcomes of pairwise and network meta‐analyses are detailed in Table S1. Olanzapine had an OR for side effects of 0.64 (95% CI 0.11–3.79) compared with enobosarm 1 mg, 0.47 (95% CI 0.08–2.71) compared with enobosarm 3 mg, 0.3 (95% CI 0.06–1.58) compared with anamorelin (50 mg), and 0.29 (95% CI 0.07–1.28) compared with anamorelin (100 mg).

### 3.4 Risk of bias

The risk of bias is presented in Figure S2, indicating the quality of all studies as acceptable. *I* ^2^ scores were 51.9% for assessing efficacy and 62.6% for assessing safety profiles of pharmacotherapy.

## 4 DISCUSSION

This study presents compelling evidence supporting the use of olanzapine, espindolol, and anamorelin in improving BW in patients with cancer cachexia through pairwise analysis. However, the network meta‐analysis did not confirm the superiority of olanzapine over espindolol or anamorelin. Regarding safety profiles, olanzapine exhibited the least side effects, followed by enobosarm and anamorelin. In addition, the network meta‐analysis did not show a significant difference for olanzapine compared with anamorelin. Espindolol showed a relatively high frequency of side effects compared to olanzapine. These results align with previous RCTs, confirming the efficacy of pharmacotherapy in mitigating weight loss among patients with cancer cachexia.^9^, ^25^, ^26^ Furthermore, the outcomes of this study underscore the potential for tailoring treatment choices based on patient background, opening avenues for personalized medicine in managing cancer cachexia. This nuanced approach to treatment selection underscores the importance of considering individual patient characteristics and disease trajectories in clinical decision‐making.

While interpreting the results of the meta‐analysis, consideration must be given to the differing inclusion criteria for olanzapine and anamorelin. For the anamorelin study, enrollment was limited to patients with NSCLC, whereas the study on olanzapine did not specify the types of cancers in participants. Furthermore, the anamorelin study adhered to the cancer cachexia criteria of weight loss of greater than 5% (involuntary weight loss) or weight loss greater than 2% in individuals already debilitated according to current BMI less than 20 kilograms/meter squared or skeletal muscle mass (sarcopenia),^27^ in contrast to the olanzapine study, which did not specify BW criteria in the published report. The characteristics of anamorelin and olanzapine differ significantly. Anamorelin is a relatively newly developed medication for cancer cachexia that comes with a caveat regarding cardiovascular side effects such as heart failure and QT prolongation, among others.^28^ On the other hand, olanzapine can be utilized as an antiemetic in oncology treatment, particularly in chemotherapy‐induced nausea and vomiting, with an administration contraindication of diabetes mellitus.^29^ These distinctive medication attributes can aid physicians in selecting the most appropriate treatment for cancer cachexia patients. Should both olanzapine and anamorelin prove intolerable, espindolol could be considered as a third option, considering tolerance to side effects.

Due to the complex mechanisms at play in cancer cachexia, combining pharmacotherapy with other treatment modalities is essential.^30^ This integrative approach embodies a holistic strategy that directly confronts the multifaceted nature of the syndrome, aiming not just at symptom management, but also at improvement of overall patient outcomes. A comprehensive treatment strategy typically encompasses pharmacotherapy to address the biological aspects of cachexia, nutritional support to counteract weight loss and malnutrition, physical therapy to preserve or enhance muscle mass and function, and psychosocial interventions to support mental and emotional well‐being.^31^, ^32^ Personalization of care is key, requiring a multidisciplinary approach that considers the overall condition of the patient, cancer type, treatment phase, and the specific manifestations of cachexia.^33^ This tailored approach ensures that interventions are not only effective, but also adaptable to the evolving needs of the patient, thereby optimizing patient care and potentially extending survival. As the understanding of the biology underlying cachexia advances, the development of more targeted therapies and refined management strategies continues, offering hope for more effective interventions in the future.

This study has several limitations. First, this meta‐analysis focused on weight gain alone as a measure of benefit in treating cachexia. Improved function and survival should also be considered, particularly since weight gain may not necessarily lead to better survival or enhanced function. However, the limited studies provided data on OS and PFS for meta‐analysis. Second, substantial heterogeneity was observed in this meta‐analysis, which may undermine the robustness of the conclusion. Third, definitions of cancer cachexia differed between studies. Fourth, no direct comparisons between different pharmacotherapies were made for cancer cachexia.

## 5 CONCLUSION

Olanzapine, anamorelin, and espindolol have demonstrated effectiveness in improving BW, although the safety profiles differ. Personalized selection of appropriate pharmacotherapies is essential in patients with cancer cachexia.

## AUTHOR CONTRIBUTIONS

**Hao Chen:** Conceptualization (lead); data curation (lead); formal analysis (lead); methodology (lead); project administration (lead); writing – original draft (lead). **Masashi Ishihara:** Conceptualization (equal); data curation (equal); formal analysis (equal). **Hiroki Kazahari:** Methodology (equal); project administration (equal); writing – original draft (equal). **Ryusuke Ochiai:** Writing – original draft (equal). **Shigeru Tanzawa:** Writing – original draft (equal). **Takeshi Honda:** Writing – original draft (equal). **Yasuko Ichikawa:** Writing – original draft (equal). **Nobuyuki Horita:** Methodology (equal); supervision (equal); validation (equal). **Hisashi Nagai:** Writing – original draft (equal). **Kiyotaka Watanabe:** Writing – original draft (equal). **Nobuhiko Seki:** Project administration (equal); writing – original draft (equal).

## FUNDING INFORMATION

No outside funding was provided.

## CONFLICT OF INTEREST STATEMENT

The authors have no conflict of interest to declare.

## Supporting information


Data S1:

## Data Availability

Raw data are available upon reasonable request to the corresponding author, nseki@med.teikyo-u.ac.jp.
