# Supplementary material for: Efficacy and safety of pharmacotherapy for cancer cachexia: A systematic review and network meta‐analysis
Source: Cancer Med. 2024 Sep 3;13(17):e70166. doi: 10.1002/cam4.70166 (PMC11369987; doi:10.1002/cam4.70166)
Supplement: Supplementary file 1 — Data S1: [file CAM4-13-e70166-s001.docx]

**Title:** Efficacy and safety of pharmacotherapy for cancer cachexia: a systematic review and network meta-analysis

**Authors:** Hao Chen^*1,2^, Masashi Ishihara^*1^, Hiroki Kazahari^1^, Ryusuke Ochiai^1^, Shigeru Tanzawa^1^, Takeshi Honda^1^, Yasuko Ichikawa^1^, Nobuhiko Horita^3^, Hisashi Nagai^4,5^, Kiyotaka Watanabe^1^, Nobuhiko Seki^1^

**Affiliations:**

^1^ Department of Oncology, Teikyo University School of Medicine, Tokyo, Japan

^2^ Department of Pulmonology, Yokohama City University Hospital, Yokohama, Japan

^3^ Department of Chemotherapy, Yokohama City University Hospital, Yokohama, Japan

^4^ Graduate School of Human and Environmental Studies, Tokai University, Tokyo Japan ^5^Ginza Phoenix Clinic, Tokyo, Japan

*H.C. and I.M. contributed equally to this article.

**Corresponding author:**

Nobuhiko Seki

Department of Oncology, Teikyo University Hospital, 2-11-1 Kaga, Itahashi, Tokyo 173-8606, Japan

E-mail: nseki@med.teikyo-u.ac.jp

Tel: 03-3964-1211

Fax: 03-3964-1211

**Supplementary materials**

**Figure S1. Flow diagram for network meta-analysis**

**
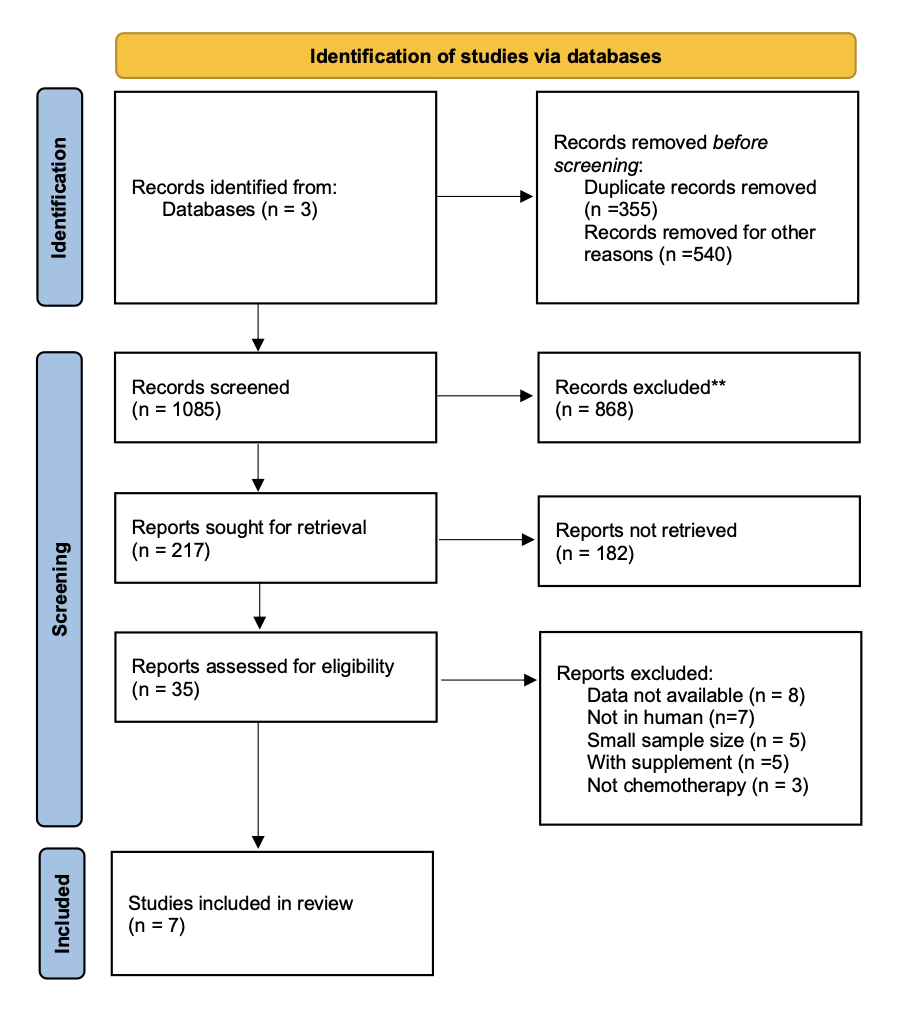
**

**Figure S2.** Rank of possibility of adverse events by each treatment based on 1000 simulations

**Figure S3.** Risk of bias

| **Table S1.** League table of pairwise and network meta-analyses | | | | | | | | | |
| --- | --- | --- | --- | --- | --- | --- | --- | --- | --- |
| Olanz |  | . | . | . | 0.26  (0.07,0.94) |  | . | . | . |
| 0.64  (0.11, 3.79) | Espin_l | 0.73  (0.21, 2.58) |  | . | 0.40  (0.12,1.38) |  | . | . | . |
| 0.47  (0.08, 2.71) | 0.73  (0.21, 2.58) | Espin_h |  | . | 0.55  (0.17,1.82) |  | . | . | . |
| 0.3  (0.06, 1.58) | 0.47  (0.09, 2.35) | 0.64  (0.13, 3.13) | Anamo_l | 1.21  (0.39, 3.73) | 0.68  (0.22,2.16) |  | . | . | . |
| 0.29  (0.07, 1.28) | 0.45  (0.11, 1.9) | 0.62  (0.15, 2.51) | 0.97  (0.34, 2.75) | Anamo_h | 0.89  (0.43,1.88) |  | . | . | . |
| 0.26  (0.07, 0.94) | 0.4  (0.12, 1.38) | 0.55  (0.17, 1.82) | 0.86  (0.3, 2.48) | 0.89  (0.43, 1.88) | place | 0.73  (0.19, 2.86) | 0.39  (0.08,1.92) | 0.28  (0.09,0.93) | 0.14  (0.01,3.14) |
| 0.19  (0.03, 1.23) | 0.29  (0.05, 1.85) | 0.4  (0.07, 2.47) | 0.63  (0.11, 3.54) | 0.65  (0.14, 3.09) | 0.73 (0.19, 2.86) | Espin_h | 0.53  (0.12,2.42) |  | . |
| 0.1  (0.01, 0.78) | 0.16  (0.02, 1.18) | 0.21  (0.03, 1.57) | 0.34  (0.05, 2.27) | 0.35  (0.06, 2.02) | 0.39 (0.08, 1.92) | 0.53 (0.12, 2.42) | Espin_l |  | . |
| 0.07  (0.01, 0.42) | 0.11  (0.02, 0.63) | 0.16  (0.03, 0.84) | 0.25  (0.05, 1.2) | 0.25  (0.06, 1.03) | 0.28 (0.09, 0.93) | 0.39 (0.06, 2.38) | 0.73 (0.1, 5.32) | Mirta |  |
| 0.04  (0, 1.05) | 0.06  (0, 1.6) | 0.08  (0, 2.15) | 0.12  (0, 3.22) | 0.12  (0.01, 3.06) | 0.14 (0.01, 3.14) | 0.19 (0.01, 5.73) | 0.36 (0.01, 11.84) | 0.49 (0.02, 13.71) | Pento |

Olan: olanzapine 2.5 mg; Espin_h espindolol 20 mg; Anamo_h: anamorelin 100 mg; Anamo_l: anamorelin 50 mg; Espin_l: espindolol 10 mg; Mirta: mirtazapine; Pento: pentoxifylline 1200 mg; Enobo_h: enobosarm 3 mg; Enobo_l: enobosarm 1 mg; Place: placebo.
